# A case of *EGFR* mutation-positive lung adenocarcinoma in which the T790M allele fraction was increased by repeated EGFR-TKI treatment

**DOI:** 10.1186/s40880-019-0413-5

**Published:** 2019-11-01

**Authors:** Hideharu Kimura, Yoshiaki Amino, Hayato Koba, Yuichi Tambo, Noriyuki Ohkura, Johsuke Hara, Takashi Sone, Kazuo Kasahara

**Affiliations:** 1 Department of Respiratory Medicine, Kanazawa University Hospital, Takara-machi 13-1, Kanazawa, Ishikawa 920-8641 Japan; 2 Regional Respiratory Symptomatology, Kanazawa University Graduate School of Medical Science, Kanazawa, Ishikawa 920-8641 Japan; 3 Respiratory Medicine, Institute of Medical, Pharmaceutical and Health Sciences, Kanazawa University Faculty of Medicine, Kanazawa, Ishikawa 920-8641 Japan

Dear editor,

The current treatment strategy for advanced non-small cell lung cancer (NSCLC) patients harboring epidermal growth factor receptor (*EGFR*) mutations involves EGFR-tyrosine kinase inhibitors (EGFR-TKIs). Several reports on EGFR-TKI resistance have shown that the T790M mutation within the *EGFR* gene is present in approximately half of the patients who develop resistance to a first- or second-generation EGFR-TKI [1, 2]. In Japan, osimertinib monotherapy is the standard therapy for patients harboring the *EGFR* T790M mutation arising after the development of resistance to a first- or second-generation EGFR-TKI [3, 4]. Herein, we report the case of a patient who underwent four tumor biopsies over the treatment course. Despite no detection of this mutation after developing resistance to the first-line EGFR-TKI, in the fourth biopsy specimen, which was collected after progression following EGFR-TKI re-challenge, *EGFR* T790M mutation was detected using the cobas^®^ EGFR mutation test (Roche Molecular Systems, Pleasanton, CA, USA). Additionally, we analyzed the T790M allele frequency (AF) in longitudinal biopsy samples obtained at four different time points using droplet digital PCR (ddPCR) (PrimePCR™, Bio-Rad Laboratories, Inc., Hercules, CA, USA). We discuss the clinical usefulness of longitudinal assessment of the *EGFR* T790M AF, especially for deciding the appropriate treatment (e.g., osimertinib) in patients with *EGFR* mutations.

## Case presentation

**Fig. 1:**
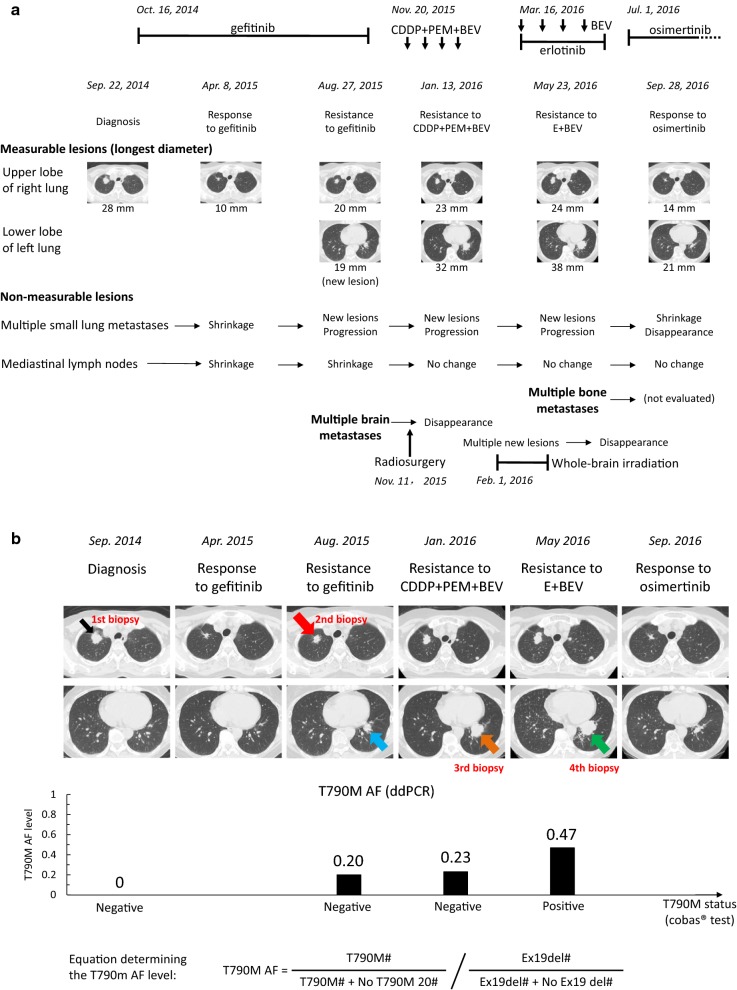
**a** Clinical course of this case. **b** Changes in chest CT images and T790M status. The upper and lower images show the upper and lower lobes, respectively. The narrow black arrow indicates the nodule in the upper lobe of the right lung at diagnosis; the first biopsy was performed in this nodule in September 2014, which was diagnosed as EGFR Ex19del-positive and T790M-negative adenocarcinoma according to the cobas^®^ test. The nodule in the upper lobe of the right lung shrank after gefitinib treatment and then increased again. The second biopsy was performed in August 2015 in the nodule indicated by the thick red arrow, which was diagnosed as EGFR Ex19del-positive and T790M-negative adenocarcinoma according to the cobas^®^ test. A new nodule appeared as a resistant tumor in the lower lobe of the left during gefitinib treatment (blue arrow), and this nodule increased after CDDP + PEM + BEV treatment. The third biopsy was performed in January 2016 in the nodule indicated by the orange arrow, which was diagnosed as EGFR Ex19del-positive and T790M-negative adenocarcinoma according to the cobas^®^ test. The mass in the lower lobe of the left lung had increased and multiple small nodules had appeared after EGFR-TKI re-challenge with E + BEV. The fourth biopsy was performed in the mass indicated by the green arrow, which was diagnosed as adenocarcinoma positive for both EGFR Ex19del and T790M according to the cobas^®^ test. The lesions improved after initiation of osimertinib treatment. The T790M AFs are shown in the graph below. T790M#, WT20#, Ex19del#, and WT19# refer to the copy numbers of the EGFR T790M mutation, no T790M in EGFR exon 20, deletion in EGFR exon 19, and no deletion in EGFR exon 19, respectively. *CT* computed tomography, *EGFR* epidermal growth factor receptor, *CDDP* cisplatin, *PEM* pemetrexed, *BEV* bevacizumab, *TKI* tyrosine kinase inhibitor, *E* erlotinib, *AF* allele frequency

A 54-year-old Japanese woman who had never smoked and had no past medical history was diagnosed at Kanazawa University Hospital (Kanazawa, Ishikawa, Japan) with stage IV *EGFR* mutation-positive (a deletion mutation in exon 19, Ex19del) lung adenocarcinoma on September 22, 2014. She had a primary nodule in the upper lobe of the right lung (Fig. 1a), multiple small metastases in both lobes, lymph node metastases in the right hilum and mediastinum but no brain metastases at diagnosis. She had worked as a school cook, an occupation seemingly unrelated to her diagnosis.

A tumor specimen was collected on the upper lobe of her right lung (Fig. 1b, first column). The patient first underwent treatment with gefitinib (250 mg daily, Iressa^®^, AstraZeneca, Cambridge, UK) starting on October 16, 2014, and achieved a partial response by April 8, 2015, according to the Response Evaluation Criteria in Solid Tumors (version 1.1) (Fig. 1b, second column). On August 27, 2015, 10 months after the initiation of gefitinib, the size of a nodule in the right lower lobe had increased and multiple small nodules were detected. On November 11, 2015, a nodule in the lower lobe of the left lung and multiple brain metastases had developed (Fig. 1a, b, third column and Additional file 1: Figure S1). Computed tomography-guided transthoracic needle biopsy of the regrowth nodule in the upper lobe of the right lung was performed on October 13, 2015. Adenocarcinoma was diagnosed, and the *EGFR* mutation status was Ex19del (no T790M) according to the cobas^®^ test. After radiosurgery was performed on the brain metastases on November 16, 2015, a combination therapy consisting of cisplatin (75 mg/m^2^, Maruko cisplatin, Yakult, Tokyo, Japan), pemetrexed (500 mg/m^2^, Alimta^®^, Eli Lilly, Indianapolis, IN) and bevacizumab (15 mg/kg, Avastin^®^, Chugai, Tokyo, Japan) (CDDP + PEM + BEV) every 3 weeks, was initiated as the second therapy starting on November 20, 2015. After four cycles of the combination therapy, the number of brain metastases and lung lesions increased on January 19, and January 13, 2016 (Fig. 1a, b, fourth column, Additional file 1: Figure S1). The patient then underwent whole-brain irradiation at a total dose of 40 Gy (2 Gy, 20 times) from February 1, 2016 to March 2, 2016, after which the growing mass in the lower lobe of the left lung was diagnosed as adenocarcinoma positive for Ex19del (no T790M) via transbronchial biopsy on February 25, 2016 (Fig. 1b, arrows). Combination therapy of erlotinib (150 mg daily, Tarceva^®^, Chugai, Tokyo, Japan) and bevacizumab (15 mg/kg, every 3 weeks) (E + BEV) was initiated as the third-line treatment (EGFR-TKI re-challenge) starting on March 16, 2016. After 2 months, multiple pulmonary nodules were observed to have increased in size, as detected by chest CT on May 23, 2016 (Fig. 1a, b, fifth column), and multiple bone metastases had newly emerged, as detected by positron emission tomography/CT on May 31, 2016. The growing nodule in the lower lobe of the left lung was diagnosed as adenocarcinoma positive for both *EGFR* Ex19del and T790M at the fourth biopsy via transbronchoscopy on June 8, 2016. The patient received osimertinib (Tagrisso^®^, 80 mg daily, AstraZeneca) monotherapy as the fourth-line treatment starting on July 1, 2016, and experienced a partial response (Fig. 1b, sixth column).

The T790M AFs in tumor tissues were calculated based on the T790M copy numbers detected by ddPCR, providing a quantitative evaluation of the AF (Fig. 1b). DNA was extracted from tumor samples collected via the four biopsies by macro-dissection. The T790M AFs levels in tumor tissues at baseline, resistance to gefitinib (first EGFR-TKI), resistance to CDDP + PEM + BEV, and resistance to E + BEV (EGFR-TKI re-challenge) were 0, 0.20, 0.23, and 0.47, respectively (Fig. 1b, bottom panel). T790M was detected at a low AF (0.20), even though no mutation was detected by the cobas^®^ test. Additionally, the T790M AF was found to be higher after progression with E + BEV (EGFR re-challenge treatment) than after progression with gefitinib.

## Discussion

In the presented case, the patient was diagnosed as *EGFR* T790M positive after the fourth biopsy but T790M was already present at a very low AF after treatment with the first EGFR-TKI (gefitinib). The T790M AF then increased after additional EGFR-TKI exposure, thus enabling detection of T790M by the cobas^®^ test. We provide the first evidence of this phenomenon by quantifying the longitudinal changes in T790M AF using ddPCR. We also found that the change in T790M AF over the course of treatment with different EGFR-TKIs influenced the results of an approved T790M mutation test that is widely used in daily care (i.e., the cobas^®^ test). In the present case, the T790M mutation was detected after the development of resistance to E + BEV (EGFR-TKI re-challenge treatment), despite no detection of this mutation was identified by the cobas^®^ test following the development of resistance to gefitinib (the first EGFR-TKI used). However, ddPCR revealed that the T790M AF was low following gefitinib treatment and then increased after E + BEV (EGFR-TKI re-challenge) treatment. Thus, the T790M allele was present at a low frequency, below the limit of detection of the cobas^®^ test, in the recurrent tumor. There are two possible explanations for the longitudinal fluctuation in the T790M AF in the present case. First, differences in treatment may have affected the sensitivity of T790M AF detection. In the present case, the T790M AF was below the detection limit of the cobas^®^ test after gefitinib treatment but subsequently increased to the detection limit after E + BEV (EGFR-TKI re-challenge) treatment. More than half of the cases of resistance to gefitinib or erlotinib have been associated with the secondary mutation T790M in *EGFR* exon 20 [1, 2]. Meanwhile, the incidence of T790M mutation following E + BEV treatment has not yet been determined. Second, the EGFR-TKI treatment duration may have affected the T790M AF. In individual prospective studies, Kawamura et al. [5] and Oya et al. [6] reported that longer-term administration of EGFR-TKIs may be a predictive marker of T790M mutation occurrence. Although CDDP + PEM + BEV was administered between the gefitinib and E + BEV treatments in the present case, no change in the T790M AF occurred during CDDP + PEM + BEV treatment: however, the T790M AF was increased after additional EGFR-TKI treatment with E + BEV. Regardless of which of the above explanations is correct, we strongly believe that EGFR-TKI treatment may influence the T790M AF. Combination of EGFR-TKIs with other treatment agents, such as angiogenesis inhibitors, and long-term EGFR-TKI treatment may increase the T790M AF. By establishing a treatment method that increases the T790M AF, second-line treatment with osimertinib to target T790M could potentially increase the survival of *EGFR* mutation-positive patients.

Repeated biopsies, but not re-biopsy following the first EGFR-TKI, may also increase the detection rate of the T790M mutation. Kuiper et al. [7] analyzed the T790M mutation status in repeated biopsy specimens obtained from 10 patients after EGFR-TKI re-challenge who did not have the T790M mutation at the time of developing resistance to first-line EGFR-TKI therapy and observed that the T790M mutation was detected in 5 of them. However, that study did not report the response to third-generation EGFR-TKIs, such as osimertinib, nor any ddPCR results. According to the T790M AF analyses in this case report, we propose that the T790M AF can be used as a predictor of response to osimertinib, and evaluating the longitudinal changes in the T790M AF under EGFR-TKI treatment may be useful for determining the subsequent treatment regimen (e.g., osimertinib) in patients with *EGFR* mutations. As reported by Ariyasu et al. [8], the effect of osimertinib is expected to be greater when the T790M AF is higher. A cutoff T790M AF is needed to predict the clinical effectiveness of osimertinib, and this is an issue to address in the near future. When the AF is below the cutoff, anti-tumor agents such as first- or second-generation EGFR-TKIs can be selected as the re-challenge treatment. If the T790M AF increases above the cutoff due to long-term EGFR-TKI treatment, osimertinib can be opted as the next treatment. Establishing a method for determining the treatment sequence based on the T790M AF is expected to improve the prognosis of patients treated with EGFR-TKIs. Additionally, osimertinib treatment resulted in longer progression-free survival than treatment with first-generation EGFR-TKIs (gefitinib or erlotinib) in treatment-naïve patients with EGFR-mutated NSCLC in a randomized phase III trial, leading to the approval of osimertinib monotherapy as first-line treatment [9]. Considering that patients are currently receiving first-line osimertinib, future studies should focus on tumor-derived T790M AF in a greater number of patients. Additionally, in clinical practice, circulating tumor DNA (ctDNA) is used as a non-invasive liquid biopsy method for *EGFR* mutation detection. Remon et al. [10] reported the efficacy of osimertinib in patients with *EGFR* T790M-positive ctDNA. They concluded that ctDNA can be used as a surrogate tissue for *EGFR* T790M testing for the purpose of deciding whether to use osimertinib as the next treatment.

In conclusion, the T790M AF of NSCLC patients receiving EGFR-TKIs was found to fluctuate during the course of treatment and influenced the results of the commonly used cobas^®^ EGFR T790M mutation test. Combination of EGFR-TKIs with other treatment agents, such as angiogenesis inhibitors, and long-term EGFR-TKI treatment may increase the level of T790M AF. Establishing a method of determining the treatment sequence based on the T790M AF may improve the prognosis of patients treated with EGFR-TKIs. We propose that multiple biopsies of tumors at different treatment stage/interval to analyze the possibility of resistance to EGFR-TKIs demonstrating TKI resistance should be performed to select the most effective treatment, such as osimertinib.

## Supplementary information

**Additional file 1: Figure S1.** Changes detected on brain MRI. After the development of resistance to gefitinib, multiple brain metastases were detected. Although the metastases disappeared after radiosurgery, new development of multiple brain metastases was detected after CDDP + PEM + BEV. The brain metastases improved after whole-brain irradiation following CDDP + PEM + BEV and have not worsened since then. Therefore, the effectiveness of EGFR-TKIs for treating brain metastases is not clear

## Data Availability

All data generated or analyzed in this study are included in the article.

## Abbreviations

NSCLC: non-small cell lung cancer
EGFR: epidermal growth factor receptor
TKI: tyrosine kinase inhibitor
AF: allele frequency
ddPCR: droplet digital PCR
Ex19del: a deletion mutation in exon 19
CDDP: cisplatin
PEM: pemetrexed
BEV: bevacizumab
E: erlotinib
ctDNA: circulating tumor DNA
