# Supplementary material for: A case of EGFR mutation-positive lung adenocarcinoma in which the T790M allele fraction was increased by repeated EGFR-TKI treatment
Source: Cancer Commun (Lond). 2019 Nov 1;39:67. doi: 10.1186/s40880-019-0413-5 (PMC6823937; doi:10.1186/s40880-019-0413-5)
Supplement: Supplementary file 1 — Additional file 1: Figure S1. Changes detected on brain MRI. After the development of resistance to gefitinib, multiple brain metastases were detected. Although the metastases disappeared after radiosurgery, new development of multiple brain metastases was detected after CDDP + PEM + BEV. The brain metastases improved after whole-brain irradiation following CDDP + PEM + BEV and have not worsened since then. Therefore, the effectiveness of EGFR-TKIs for treating brain metastases is not clear [file 40880_2019_413_MOESM1_ESM.pptx]

## Slide 1
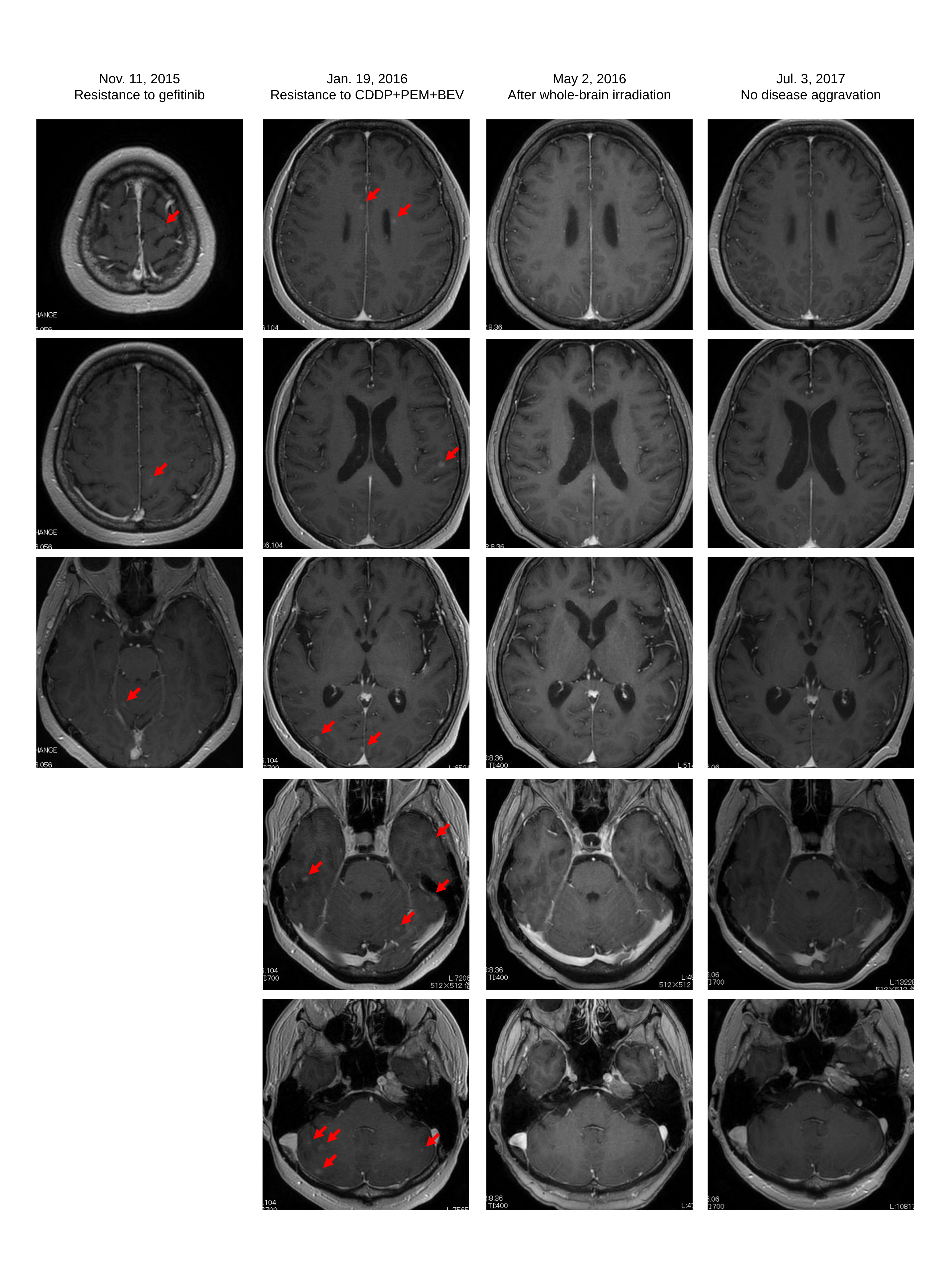

Nov. 11, 2015
Resistance to gefitinib
Jan. 19, 2016
Resistance to CDDP+PEM+BEV
May 2, 2016
After whole-brain irradiation
Jul. 3, 2017
No disease aggravation
